# Accelerated tibia fracture healing in traumatic brain injury in accordance with increased hematoma formation

**DOI:** 10.1186/s12891-022-06063-5

**Published:** 2022-12-20

**Authors:** Dong Woo Shim, Hyunjoo Hong, Kwang-Chun Cho, Se Hwa Kim, Jin Woo Lee, Seung-Yong Sung

**Affiliations:** 1 Department of Orthopedic Surgery, International St. Mary’s Hospital, Catholic Kwandong University College of Medicine, 25, Simgok-ro 100beon-gil, Seo-gu, Incheon, 22711 South Korea; 2 Department of Radiology, Severance Health Check-up, 10, Tongil-ro, Jung-gu, Seoul, Republic of Korea; 3 Department of Neurosurgery, Yongin Severance Hospital, Yonsei University College of Medicine, Severance Hospital 363, Dongbaekjukjeon-daero, Giheung-gu, Yongin-si, Gyeonggi-do Republic of Korea; 4 Department of Internal Medicine, International St. Mary’s Hospital, Catholic Kwandong University College of Medicine, 25, Simgok-ro 100beon-gil, Seo-gu, Incheon, 22711 South Korea; 5 Department of Orthopaedic Surgery, Severance Hospital, Yonsei University College of Medicine, 50 Yonsei-ro, Seodaemun-gu, Seoul, 03722 South Korea

**Keywords:** Traumatic brain injury, Fracture healing, Callus formation, Hematoma

## Abstract

**Background:**

Traumatic brain injury (TBI) has been known to accelerate bone healing. Many cells and molecules have been investigated but the exact mechanism is still unknown. The neuroinflammatory state of TBI has been reported recently. We aimed to investigate the effect of TBI on fracture healing in patients with tibia fractures and assess whether the factors associated with hematoma formation changed more significantly in the laboratory tests in the fractures accompanied with TBI.

**Methods:**

We retrospectively investigated patients who were surgically treated for tibia fractures and who showed secondary bone healing. Patients with and without TBI were divided for comparative analyses. Radiological parameters were time to callus formation and the largest callus ratio during follow-up. Preoperative levels of complete blood count and chemical battery on admission were measured in all patients. Subgroup division regarding age, gender, open fracture, concomitant fracture and severity of TBI were compared.

**Results:**

We included 48 patients with a mean age of 44.9 (range, 17–78), of whom 35 patients (72.9%) were male. There were 12 patients with TBI (Group 1) and 36 patients without TBI (Group 2). Group 1 showed shorter time to callus formation (*P* <  0.001), thicker callus ratio (*P* = 0.015), leukocytosis and lymphocytosis (*P* ≤ 0.028), and lower red blood cell counts (RBCs), hemoglobin, and hematocrit (P <  0.001). Aging and severity of TBI were correlated with time to callus formation and callus ratio (*P* ≤ 0.003) while gender, open fracture, and concomitant fracture were unremarkable.

**Conclusion:**

Tibia fractures with TBI showed accelerated bone healing and superior measurements associated with hematoma formation (lymphocytes, RBCs, hemoglobin, hematocrit). Promoted fracture healing in TBI was correlated with the enhanced proinflammatory state.

**Level of evidence:**

III, case control study.

## Background

After debatable results of traumatic brain injury (TBI) and accelerated fracture healing of earlier studies, many preclinical and clinical following studies supported that TBI is associated with rapid bone healing [1–4]. Various materials from cytokine and growth factors to genes and hormones have been investigated as candidate substances associated with TBI but the mechanism has not been fully understood [4–6].

Fracture healing comprises of inflammatory phase, fibrovascular phase, bone formation phase, and remodeling phase. Inflammatory phase starts with fibrin-rich hematoma formation from shearing of intracortical, endosteal, and periosteal vessels [7–9]. Chemokines from activated platelets within the hematoma accelerate the migration of neutrophils and macrophages to the fracture site [10]. These cells remove devitalized tissue around the fracture site, promote recruiting inflammatory cells and successively progenitor cells from the bone marrow, periosteum, soft tissue and systemic circulation [11, 12].

Previous clinical studies have shown promoted osteogenic effect in TBI, but little has been studied through blood test results. Cadosch et al. investigated the human fetal osteoblastic (hFOB) cell and other biochemical markers including C-reactive protein, alkaline phosphatase, calcium, phosphate, and parathyroid hormone in long bone fracture patients with and without TBI. They showed higher proliferation of hFOB cells in TBI group whereas other biochemical markers showed unremarkable results [1]. A recent preclinical study reported that TBI alters the local neuroinflammatory state to accelerate early fracture healing [13]. They showed strong positive relationship between hematoma formation and fracture healing.

In the present study, we were to reaffirm if TBI accelerated tibia fracture healing. Moreover, we assessed whether the factors associated with hematoma formation changed more significantly in the laboratory tests in the fractures accompanied with TBI.

## Methods

We retrospectively investigated 512 consecutive patients who were treated for tibia fractures from February 2014 to January 2020 in our institution. We included patients between 17 and 80 years of age, who underwent plate fixation or intramedullary nailing for comminuted fractures and showed secondary bone healing. Patients with isolated intraarticular fracture healed without callus formation, fixated with screws only, severe open fracture (Gustilo-Anderson type ≥ III), and underlying conditions that could impair bone healing (diabetes, cancer, organ transplantation, chronic renal failure, prolonged use of steroid, infection and etc.) were excluded. Brain injury was evaluated with Glasgow Coma Scale (GCS) when patients showed any kind of neurological impairments and brain computed tomography (CT) was checked subsequently. The type and extent of brain hemorrhage was assessed via CT scans and were scored according to Marshall classification system by a radiologist; category 1, no intracranial pathology seen on CT; category 2, cisterns present with midline shift of 0–5 mm and/or lesions/densities present, no high or mixed density lesions > 25 cm^3^, may include bone fragments and foreign bodies; category 3, cisterns compressed or absent with midline shift of 0–5 mm, no high or mixed density lesions > 25 cm^3^; category 4, midline shift > 5 mm, no high or mixed density lesions > 25 cm^3^; category 5, any lesion surgically evacuated; category 6, high or mixed density lesion > 25 cm^3^, not surgically evacuated [14]. Neurosurgeons decided whether to evacuate the hematoma depending on the amount of intracranial hemorrhage. Patients were divided into two groups if they accompanied moderate to severe TBI (GCS ≤ 12) or not for the case-control study.

We included 48 patients with a mean age of 44.9 (range, 17–78), of whom 35 patients (72.9%) were male. There were 12 patients with TBI (Group 1) and 36 patients without TBI (Group 2). In Group 1, mean GCS was 6.2 (range, 3–12); there were 4 patients with subdural hemorrhage, 2 patients with epidural hemorrhage and 6 patients with combined injuries (3 with intracerebral and subarachnoid, 2 with subdural and subarachnoid, 1 with intracerebral, subarachnoid, and intraventricular hemorrhages). None of them expired during follow-up (mean 32.4 months, range 12–60). Four patients underwent burr hole trephination. Six patients were grade 2, 2 patients were grade 3, and 4 patients were grade 5 according to the Marshall classification of TBI. Patient characteristics between the groups are summarized in Table 1.

**Table 1:** Patient characteristics of tibia fractures with and without traumatic brain injury

	Group 1 (*N* = 12)	Group 2 (*N* = 36)	*P* value
Age	41.5 (14.2)	46.1 (15.4)	0.294
Gender (Male, %)	10 (83.3)	25 (69.4)	0.469
AO classification (*n*, %)			0.130
42B	–	3 (8.3)	
42C	–	1 (2.8)	
43A	6 (50)	21 (58.3)	
43C	6 (50)	11 (30.6)	
Time to operation (days)	10.3 (4.0)	4.8 (4.1)	< 0.001*
Open fracture (Yes, %)	–	4 (11.1)	0.560
External fixation (Yes, %)	2 (16.7)	10 (27.8)	0.703
Concomitant fracture lesion (Yes, %)	12 (100)	4 (11.1)	0.001*
Skull	10	–	
Spine	6	–	
Facial bone	5	–	
Rib	4	–	
Clavicle	2	1	
Forearm	3	1	
Femur	2	2	
Contralateral lower leg	–	2	

Data are mean (SD) or *n* (%)

*Statistically significant (*P* <  0.05)

### Radiological and clinical outcomes

**Fig. 1:**
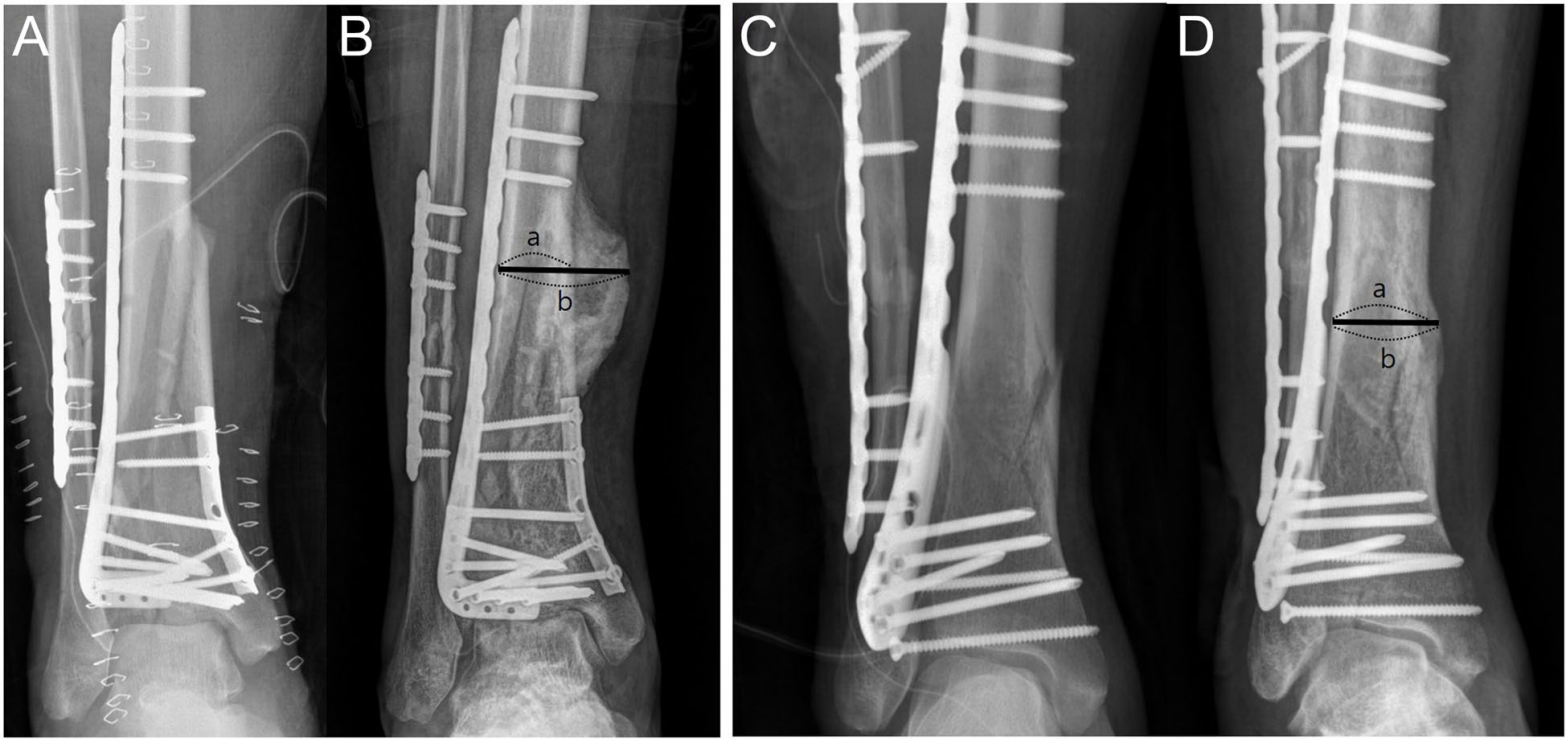
**A**, **B** AP radiographs of a patient with traumatic brain injury at immediate post-operative and at 6 months follow-up. **C**, **D** AP radiographs of a patient without traumatic brain injury at immediate post-operative and at 3 months follow up. Callus ratio measuring method by Spencer = b/a

The patient data included general demographic data, tibia fracture type, concomitant fracture, performance of external fixation or not, and presence of open fracture. Short leg splint was applied to all patients, and range of motion and non-weightbearing were maintained for 6 weeks. Patients were assessed at 6 weeks, 3 months, 6 months, and 12 months postoperatively with anteroposterior, lateral, and both oblique radiographs. Main radiologic outcome measures included time to callus formation and the widest callus ratio during follow-up. Time to callus formation was defined as the first appearance of the callus from the date of trauma either on anteroposterior, lateral, or both oblique radiographs and the callus ratio was measured using the method previously described by Spencer (Fig. 1.) [15]. Two independent authors assessed these radiologic parameters and a 1-week washout period was implemented before additional measurement.

Laboratory tests were obtained from all patients at the time visiting the hospital. The levels of complete blood count (CBC), chemical battery including alkaline phosphatase (ALP), calcium, phosphate and C-reactive protein (CRP) were measured in all collected samples. Calcium and phosphate can trigger the deposition of calcium phosphate crystal in the osteoid [16–18]. ALP is secreted by osteoblast and triggers the mineralization of the osteoid [18, 19]. CRP has been known to be negatively associated with bone marrow density [19, 20].

### Statistical analyses

Patient characteristics are presented as mean (SD) or count (percentage). Statistical analysis was performed using the commercial software SPSS (version 21.0; IBM Corp, Armonk, NY). Power analysis with two tail test was performed using G power software (Ver 3.1.9.4, Germany). Mann–Whitney test, Fisher’s exact test, and linear by linear association were used for statistical analyses to compare the two groups. Correlations among the radiologic and laboratory parameters with age were examined using the Spearman correlation coefficient test. Spearman’s rho was interpreted as little (± <  0.3), low (± 0.3–0.5), moderate (± 0.5–0.7), high (± 0.7–0.9), and very high (± > 0.9) [21]. A *P* value of < 0.05 was considered statistically significant. The intraclass correlation coefficient (ICC) was used to determine the intraobserver and interobserver agreement. All ICC were interpreted as poor (< 0.2), fair (0.2–0.4), moderate (0.4–0.6), good (0.6–0.8), and very good (0.8–1.0) [22].

## Results

All patients obtained bone union within 12 months. Group 1 showed shorter time to callus formation (*P* <  0.001) and thicker callus ratio (*P* = 0.015) than Group 2. In addition, Group 1 had higher WBC (*P* = 0.028) and lymphocyte count (*P* = 0.025), and lower red blood cell counts (RBCs), hemoglobin, hematocrit (P <  0.001) compared to Group 2. Calcium level was significantly lower whereas phosphate was significantly higher in Group 1. Other laboratory markers were unremarkable. (Table 2) Time to callus formation and callus ratio showed all good reliability between intraobserver and interobserver (0.92 (95% confidence interval (CI), 0.82–0.97) and 0.82 (95% CI, 0.58–0.92)/ 0.82 (95% CI, 0.62–0.93) and 0.84 (95% CI, 0.67–0.94), respectively).

**Table 2:** Comparisons of radiological outcomes and laboratory tests between groups

	Group 1 (*N* = 12)	Group 2 (*N* = 36)	*P* value
**Radiological outcomes**			
Time to callus (days)	17.5 (9.2)	93.4 (71.8)	< 0.001*
Callus ratio	1.4 (0.2)	1.2 (0.1)	0.015*
**Laboratory tests**			
WBC (×10^3^/ul)	13.0 (2.0)	10.3 (4.1)	0.028*
Monocytes (× 10^3^/ul)	0.8 (0.4)	0.7 (0.2)	0.317
Lymphocytes (×10^3^/ul)	4.1 (2.0)	2.4 (1.0)	0.025*
Neutrophils (×10^3^/ul)	7.8 (3.1)	7.8 (4.8)	0.475
RBC (×10^6^/ul)	4.1 (0.2)	4.6 (0.5)	< 0.001*
Hb (g/dl)	12.6 (0.8)	14.4 (1.6)	< 0.001*
Hct (%)	37.3 (2.2)	42.4 (4.0)	< 0.001*
MCH (pg)	31.0 (1.4)	31.1 (1.7)	0.849
CRP (mg/l)	0.9 (0.8)	2.3 (4.0)	0.153
ALP (U/l)	93.5 (33.4)	78.0 (22.0)	0.234
Ca (mg/dl)	**8.3 (0.4)**	**8.9 (0.5)**	0.001*
*P* (mg/dl)	**4.0 (1.0)**	**3.2 (0.7)**	0.002*

Data are mean (SD)

*WBC* white blood cell, *RBC* red blood cell, *Hb* hemoglobin, *Hct* hematocrit, *MCH* mean corpuscular hemoglobin, *CRP* C-reactive protein, *ALP* alkaline phosphatase, *Ca* calcium, *P* phosphate

*Statistically significant (*P* < 0.05)

To clarify the effects of open fracture, gender, and other concomitant fracture on the callus formation, we divided the patients into two groups according to each nominal scale and compared them using Fisher’s exact test. There were no significant differences in radiological outcomes among groups divided by open fracture, gender, and concomitant fracture. RBC profiles were significantly higher in groups with open fracture and male. WBC profile and ALP tended to be higher in the male group (Table 3).

**Table 3:** Comparisons of radiological outcomes and laboratory tests between groups regarding open fracture, gender, and concomitant fracture

	Whole groups						Group 2 only		
	Without open fracture (*n* = 44)	With open fracture (*n* = 4)	*P* value	Male (*n* = 35)	Female (*n* = 13)	*P* value	Without concomitant fracture (*n* = 32)	With concomitant fracture (*n* = 4)	*P* value
**Radiological outcomes**									
Time to callus (days)	75.1 (71.6)	81.3 (71.6)	0.900	75.3 (75.2)	76.5 (60.8)	0.651	95.0 (74.7)	77.7 (34.5)	0.366
Callus ratio	1.2 (0.2)	1.2 (0.0)	0.375	1.3 (0.2)	1.2 (0.1)	0.112	1.2 (0.1)	1.2 (0.1)	0.827
**Laboratory tests**									
WBC (×10^3^/ul)	10.5 (3.6)	13.2 (5.8)	0.585	11.6 (3.6)	8.6 (3.5)	0.055	10.1 (4.2)	11.6 (4.1)	0.208
Monocytes (×10^3^/ul)	0.7 (0.3)	0.8 (0.4)	0.577	0.8 (0.3)	0.5 (0.2)	0.007*	0.6 (0.2)	0.7 (0.3)	0.716
Lymphocytes (×10^3^/ul)	2.9 (1.6)	2.4 (0.7)	0.986	3.2 (1.6)	1.9 (0.6)	0.006*	2.4 (0.9)	3.0 (2.3)	0.790
Neutrophils (×10^3^/ul)	7.6 (4.3)	9.8 (5.4)	0.375	8.0 (4.1)	7.1 (5.1)	0.302	6.9 (4.0)	7.7 (4.6)	0.292
RBC (×10^6^/ul)	4.5 (0.5)	5.0 (0.3)	0.026*	4.6 (0.5)	4.2 (0.3)	0.010*	4.6 (0.5)	4.7 (0.5)	0.716
Hb (g/dl)	13.7 (1.5)	16.2 (1.1)	0.003*	14.4 (1.7)	12.8 (0.9)	0.002*	14.4 (1.5)	14.1 (2.2)	0.511
Hct (%)	40.5 (4.2)	46.5 (2.7)	0.007*	42.2 (4.4)	38.1 (2.7)	0.002*	42.6 (3.9)	41.3 (5.5)	0.610
MCH (pg)	30.8 (1.5)	32.4 (1.7)	0.063	31.2 (1.6)	30.2 (1.1)	0.043*	31.2 (1.6)	29.8 (1.7)	0.248
CRP (mg/l)	2.1 (3.7)	0.9 (0.4)	0.985	1.4 (1.6)	3.6 (6.1)	0.893	2.5 (4.2)	0.6 (0.2)	0.248
ALP (U/l)	83.7 (27.0)	78.8 (16.2)	0.925	88.2 (27.3)	70.4 (18.2)	0.048*	78.8 (22.0)	69.7 (25.3)	0.610
Ca (mg/dl)	8.7 (0.6)	8.9 (0.6)	0.762	8.8 (0.6)	8.8 (0.5)	0.989	9.0 (0.5)	8.2 (0.5)	0.037*
P (mg/dl)	3.5 (0.8)	2.9 (0.8)	0.227	3.4 (1.0)	3.6 (0.5)	0.328	3.2 (0.7)	3.4 (0.7)	0.657

Data are mean (SD)

*WBC* white blood cell, *RBC* red blood cell, *Hb* hemoglobin, *Hct* hematocrit, *MCH* mean corpuscular hemoglobin, *CRP* C-reactive protein, *ALP* alkaline phosphatase, *Ca* calcium, *P* phosphate

*Statistically significant (*P* < 0.05)

In addition, we intended to assess the effects of age, number of intracranial hemorrhagic lesion, and Marshall classification on the outcomes. Age showed low negative correlation with callus ratio. As number of intracranial hemorrhagic lesion increased, callus ratio and WBC count significantly decreased with moderate correlation (*P* = 0.003 and *P* = 0.006, respectively). GCS tended to be negatively correlated with callus ratio but was not statistically significant (*P* = 0.076). Marshall classification positively correlated with time to callus, hemoglobin, hematocrit, and MCH whereas it was negatively correlated with calcium and phosphate. The correlation between Marshall classification and radiological and laboratory parameters were moderate to very high. (Table 4).

**Table 4:** Spearman’s correlation tests of age, number of traumatic brain injuries, and Marshall classification with outcomes and of radiological outcome with laboratory tests

	Whole groups						Group 1 only					
	Rho (age)	*P* value	Rho (time to callus)	*P* value	Rho (callus ratio)	*P* value	Rho (number of traumatic brain injuries)	*P* value	Rho (GCS)	*P* value	Rho (Marshall classification)	*P* value
**Radiological outcomes**												
Time to callus (days)	−0.088	0.553	–		−0.089	0.549	0.297	0.348	−0.164	0.593	0.939	< 0.001*
Callus ratio	−0.458	0.001*	−0.089	0.549	–		−0.772	0.003*	−0.508	0.076	−0.154	0.632
**Laboratory tests**												
WBC (×10^3^/ul)	− 0.059	0.692	− 0.109	0.460	0.186	0.204	−0.741	0.006*	− 0.073	0.814	0.123	0.702
Monocytes (× 10^3^/ul)	− 0.107	0.469	− 0.129	0.381	0.236	0.106	−0.093	0.775	−0.486	0.092	−0.062	0.849
Lymphocytes (×10^3^/ul)	−0.062	0.676	−0.169	0.252	0.199	0.175	−0.031	0.924	0.106	0.730	−0.031	0.924
Neutrophils (×10^3^/ul)	0	1.000	0.021	0.888	0.022	0.882	−0.278	0.382	−0.017	0.957	0.123	0.702
RBC (×10^6^/ul)	−0.185	0.209	0.441	0.002**	−0.177	0.230	−0.063	0.847	0.989	< 0.001**	−0.313	0.322
Hb (g/dl)	−0.101	0.495	0.442	0.002**	−0.104	0.483	−0.123	0.702	0.464	0.110	0.617	0.033*
Hct (%)	−0.104	0.481	0.465	0.001**	−0.129	0.384	−0.123	0.702	0.464	0.110	0.617	0.033*
MCH (pg)	0.206	0.159	0.009	0.950	0.058	0.696	0.216	0.500	−0.390	0.188	0.925	< 0.001**
CRP (mg/l)	0.120	0.417	−0.015	0.921	−0.126	0.395	0.185	0.565	−0.291	0.336	−0.370	0.236
ALP (U/l)	−0.025	0.864	−0.283	0.052	−0.064	0.666	0.062	0.849	0.078	0.800	−0.401	0.196
Ca (mg/dl)	−0.153	0.315	0.345	0.020*	−0.373	0.012*	−0.204	0.526	0.537	0.059	−0.751	0.005**
P (mg/dl)	−0.177	0.245	−0.492	0.001**	0.180	0.237	−0.278	0.382	−0.156	0.610	−0.833	0.001**

*GCS* Glasgow Coma Scale, *WBC* white blood cell, *RBC* red blood cell, *Hb* hemoglobin, *Hct* hematocrit, *MCH* mean corpuscular hemoglobin, *CRP* C-reactive protein, *ALP* alkaline phosphatase, *Ca* calcium, *P* phosphate

*Statistically significant (*P* < 0.05)

**Statistically significant (*P* < 0.01)

When we focused on the correlation between radiological outcomes and laboratory test, the two radiological outcomes had no significant correlation with each other (Spearman’s rho = − 0.089, *P* = 0.549). Time to callus formation was positively correlated with RBC profile except for MCH and calcium whereas it was negatively correlated with phosphate significantly. On the contrary, callus ratio was negatively correlated with calcium level (Table 4).

## Discussion

We reaffirmed that TBI have accelerated callus formation and fracture healing in patients with tibia fractures. Time to bridging callus formation and callus ratio were significantly superior in Group 1 despite the lower calcium level. Leukocytosis and lymphocytosis were predominant and RBC profiles including hemoglobin and hematocrit were lower in Group 1. Open fracture, gender, GCS, and presence of concomitant fracture did not show significant differences in radiological outcomes. Increasing age and number of intracranial hemorrhagic lesion were negatively correlated with callus ratio. Higher Marshall classification category showed very strongly positive correlation with time to callus formation.

Garland and Dowling reported absent correlation between TBI and accelerated tibial fracture healing as a pioneer study [23]. However, it contained mixed cohort of patients including severe open fractures and various surgical or conservative treatment modalities that could have made it inconclusive. Following clinical studies showed an obvious osteogenic effect of TBI from long bones to flat bones [1–3, 24, 25]. However, few factors have been investigated as candidate substances to explain the phenomenon such as runt-related transcription factor 2, serine protease 7, cathepsin K, and hFOB1.19 cell line [1, 24]. Recently, Morioka et al. reported a neuroinflammatory response in polytrauma with TBI in rodent model [13]. They showed that hematoma formation inferred from systemic lymphocytes, RBCs, hemoglobin and hematocrit was strongly positively related with fracture healing using multivariate principal component analysis. The first stage of bone healing is the inflammatory phase. The inflammatory phase is mainly mediated by fracture hematoma consisted of blood cells, mesenchymal stem cells, fibroblasts and etc., and can last for about 5 days [4, 7]. These cells promote gathering of inflammatory cells via release of pro-inflammatory cytokines such as tumor necrosis factor-alpha, interleukins 1 and 6, and subsequent growth factors [11, 12, 26]. A closer look of the Morioka’s study reveals that TBI additional to tibia fracture showed increased WBCs, monocytes, and lymphocytes and decreased RBC profiles compared to fracture only after 5 days from injury [13]. These changes gradually recovered to normal range after 15 days from injury. Our results are consistent with results of the previous studies in that Group 1 showed significant elevation of WBC and lymphocyte (*P* ≤ 0.028) and decrease of RBC, hemoglobin, and hematocrit (*P* <  0.001) at admission although there was lack of further laboratory tests. Larger hematomas in Group 1 might have accelerated the proinflammatory response to secondary bone healing.

Moderate to severe TBI (GCS ≤ 12) is well known to cause pituitary or hypothalamic dysfunction [27]. Yang et al. showed promoted callus formation in the fracture with TBI group as well. However, when they subdivided the TBI group to GCS ≤ 8 and GCS > 8, there was no significant differences in time to callus formation and callus thickness (*P* = 0.521, *P* = 0.153) [3]. Several hormones such as leptin, prolactin, calcitonin-gene-related peptide from cerebral dysfunction and damage to blood brain barrier are believed to be the possible factors of accelerated bone healing in TBI despite that accurate mechanisms remain uncertain [4, 28–37]. Morioka et al. revealed inverse correlation of fracture callus with brain lesion by analyzing total lesion volume and gross lesion area in TBI [13]. Cadosch et al. reported a negative linear relationship between GCS and callus ratio [1]. They showed that GCS was correlated with callus ratio (*P* <  0.05), time to union (*P* = 0.04), and proliferation rate of hFOB cells after 6 hours from injury (*P* = 0.03). Similarly, this study also showed a significantly negative correlation between number of intracranial hemorrhagic lesion and callus ratio (Spearman’s rho = − 0.772, *P* = 0.003), and a negative correlation tendency between GCS and callus ratio (Spearman’s rho = − 0.508, *P* = 0.076). Interestingly, Marshall classification showed a very highly positive correlation with time to callus formation despite its negative correlation with calcium and phosphate levels (Spearman’s rho = 0.939, *P* <  0.001). Marshall classification places patients into one of six categories of increasing severity based on the findings on non-contrast brain CT scan [14]. It is primarily concerned with degree of swelling and presence and size of hemorrhage. Higher categories have worse prognosis and survival. Thus, it might be a more accurate assessment of suppression of brain function; therefore, it could be more related to callus formation. Following study of relationship between Marshall classification and bone healing in TBI with larger cohort would help assess the role of brain and estimate the accelerated fracture healing in TBI.

Aging showed a significantly negative correlation with callus ratio in this study (Spearman’s rho = − 0.458, *P* = 0.001). Increasing age has been well known to negatively affect the cellular and molecular processes of fracture healing throughout all phases [38, 39]. Intrinsic changes in stem cell population and microenvironmental changes that alter the biological activity of progenitor cells are the two aspects to potentially affect tissue regeneration.

The two radiological outcomes of time to callus formation and callus ratio did not significantly correlate with each other (Spearman’s rho = − 0.089, *P* = 0.549). Interestingly, time to callus formation was positively correlated with higher RBC profiles (Spearman’s rho = 0.441–0.465, *P* ≤ 0.002). Rapid callus formation in general could be more related to the number of RBCs capable of exchanging oxygen and waste despite the low RBC profile in group 1 in this study. These conflicting results might indicate that several mechanisms including accelerated hematoma and brain dysfunction have blended effects in promoting fracture healing. Further study with serial laboratory tests would be helpful in distinguishing their effects. Male gender showed significantly superior monocytes, lymphocytes, RBC profiles, and ALP compared to female gender. However, there was no significant differences in the two radiological outcomes between each other. It is comparable to the results of a previous study in which there was no gender difference in fracture healing [3]. Presence of open fracture below Gustilo type II did not significantly affect any radiological and laboratory tests.

This study has several limitations. First, despite that all patients in both groups were evaluated with the same postoperative follow-up protocol and were intended to be involved thoroughly, this study was a retrospective study which might have resulted in a possible selection bias. Second, the study population was relatively small, which could have decreased the statistical power of the results. Fortunately, however, the actual power was measured at about 95.2% due to significant differences in results between the groups. We could have derived a more significant categories that might have correlated with each other such as GCS. Third, the radiologic outcome measurements used in the present study would not be the latest methods such as Radiographic Union Score for Tibial fractures (RUST) [40]. The reason is that this study included both patients who underwent plate fixation or nailing for tibia fracture, thus, using RUST that has been validated only for nailing might have raised additional controversy. At last, all patients lacked pre-trauma laboratory tests which could be the reference points for analyzing the lower level of hemoglobin. In addition, there is a weak point that low level of hemoglobin contributed exclusively to the formation of fracture hematoma. However, factors which might have affected the difference of hemoglobin level such as aging and gender were not significantly different between the groups. A previous preclinical study has treated the low level of hemoglobin as the hematoma formation [13]. We believe this study is a pilot preliminary study focusing on systemic laboratory analyses in accordance with TBI in tibia fracture. Following studies regarding these factors can derive further relationship between proinflammatory response and accelerated fracture healing in TBI.

## Conclusion

Tibia fractures with TBI showed accelerated bone healing and superior measurements associated with hematoma formation (lymphocytes, RBCs, hemoglobin, hematocrit). Promoted fracture healing correlated with the promoted proinflammatory state.

## Data Availability

The datasets generated and analyzed during the current study are not publicly available but are available from the corresponding author on reasonable request.

## Abbreviations

TBI: Traumatic brain injury
hFOB: Human fetal osteoblastic
GCS: Glasgow Coma Scale
CT: Computed tomography
CBC: Complete blood count
ALP: Alkaline phosphatase
CRP: C-reactive protein
WBC: White blood cell
RBC: Red blood cell
MCH: Mean corpuscular hemoglobin

## References

[1] Cadosch D, Gautschi OP, Thyer M, Song S, Skirving AP, Filgueira L, Zellweger R (2009). Humoral factors enhance fracture-healing and callus formation in patients with traumatic brain injury. J Bone Joint Surg Am.

[2] Giannoudis PV, Mushtaq S, Harwood P, Kambhampati S, Dimoutsos M, Stavrou Z, Pape HC (2006). Accelerated bone healing and excessive callus formation in patients with femoral fracture and head injury. Injury..

[3] Yang TY, Wang TC, Tsai YH, Huang KC (2012). The effects of an injury to the brain on bone healing and callus formation in young adults with fractures of the femoral shaft. J Bone Joint Surg Br..

[4] Hofman M, Koopmans G, Kobbe P, Poeze M, Andruszkow H, Brink PR, Pape HC (2015). Improved fracture healing in patients with concomitant traumatic brain injury: proven or not?. Mediat Inflamm.

[5] Morley J, Marsh S, Drakoulakis E, Pape HC, Giannoudis PV (2005). Does traumatic brain injury result in accelerated fracture healing?. Injury..

[6] Huang H, Cheng WX, Hu YP, Chen JH, Zheng ZT, Zhang P (2018). Relationship between heterotopic ossification and traumatic brain injury: why severe traumatic brain injury increases the risk of heterotopic ossification. J Orthop Translat.

[7] Hellwinkel JE, Miclau T, Provencher MT, Bahney CS, Working ZM (2020). The life of a fracture: biologic progression, healing gone awry, and evaluation of union. JBJS Rev.

[8] Citak C, Kayali C, Ozan F, Altay T, Karahan HG, Yamak K (2019). Lateral locked plating or dual plating: a comparison of two methods in simple Bicondylar Tibial plateau fractures. Clin Orthop Surg.

[9] Shim DW, Choi E, Park YC, Shin SC, Lee JW, Sung SY. Comparing bilateral feet computed tomography scans can improve surgical decision making for subtle Lisfranc injury. Arch Orthop Trauma Surg. 2021. 10.1007/s00402-021-04182-7. 10.1007/s00402-021-04182-7 34599354

[10] Bolander ME (1992). Regulation of fracture repair by growth factors. Proc Soc Exp Biol Med.

[11] Loi F, Cordova LA, Pajarinen J, Lin TH, Yao Z, Goodman SB (2016). Inflammation, fracture and bone repair. Bone..

[12] Baht GS, Vi L, Alman BA (2018). The role of the immune cells in fracture healing. Curr Osteoporos Rep..

[13] Morioka K, Marmor Y, Sacramento JA, Lin A, Shao T, Miclau KR, Clark DR, Beattie MS, Marcucio RS, Miclau T (2019). Differential fracture response to traumatic brain injury suggests dominance of neuroinflammatory response in polytrauma. Sci Rep.

[14] Marshall LF, Marshall SB, Klauber MR, Van Berkum CM, Eisenberg H, Jane JA, Luerssen TG, Marmarou A, Foulkes MA (1992). The diagnosis of head injury requires a classification based on computed axial tomography. J Neurotrauma.

[15] Spencer RF (1987). The effect of head injury on fracture healing. A quantitative assessment. J Bone Joint Surg Br.

[16] Eschler A, Roepenack P, Herlyn PK, Roesner J, Martin H, Vollmar B, Mittlmeier T, Gradl G (2015). Intrabody application of eptotermin alpha enhances bone formation in osteoporotic fractures of the lumbar spine; however, fails to increase biomechanical stability - results of an experimental sheep model. Growth Factors.

[17] Ciosek Ż, Kot K, Kosik-Bogacka D, Łanocha-Arendarczyk N, Rotter I. The effects of calcium, magnesium, phosphorus, fluoride, and Lead on bone tissue. Biomolecules. 2021;11(4). 10.3390/biom11040506. 10.3390/biom11040506 PMC8066206 33800689

[18] Anaraki N, Beyraghi AH, Raisi A, Davoodi F, Farjanikish G, Sadegh AB (2021). The effect of aqueous extract of Prunus dulcis on tibial bone healing in the rabbit. J Orthop Surg Res.

[19] Chen Z, Xie L, Xu J, Lin X, Ye J, Shao R, Yao X (2021). Changes in alkaline phosphatase, calcium, C-reactive protein, D-dimer, phosphorus and hemoglobin in elderly osteoporotic hip fracture patients. Ann. Palliat Med.

[20] Kiran DN, Desai R (2012). Estimation of C-reactive protein associated with mandibular fracture. J Maxillofac Oral Surg.

[21] Rovai AP, Baker JD, Ponton MK. Social science research design and statistics: a practitioner's guide to research methods and IBM SPSS. Virginia: Watertree Press LLC; 2013.

[22] Altman DG. Practical statistics for medical research. London and New York: Chapman and Hall; 1991.

[23] Garland DE, Toder L (1980). Fractures of the tibial diaphysis in adults with head injuries. Clin Orthop Relat Res.

[24] Gautschi OP, Cadosch D, Frey SP, Skirving AP, Filgueira L, Zellweger R (2009). Serum-mediated osteogenic effect in traumatic brain-injured patients. ANZ J Surg.

[25] Huang W, Li Z, Li Z, Yang R (2012). Does traumatic brain injury result in accelerated mandibular fracture healing?. J Oral Maxillofac Surg.

[26] Kolar P, Schmidt-Bleek K, Schell H, Gaber T, Toben D, Schmidmaier G, Perka C, Buttgereit F, Duda GN (2010). The early fracture hematoma and its potential role in fracture healing. Tissue Eng Part B Rev.

[27] Javed Z, Qamar U, Sathyapalan T (2015). Pituitary and/or hypothalamic dysfunction following moderate to severe traumatic brain injury: current perspectives. Indian J Endocrinol Metab.

[28] Ducy P, Amling M, Takeda S, Priemel M, Schilling AF, Beil FT, Shen J, Vinson C, Rueger JM, Karsenty G (2000). Leptin inhibits bone formation through a hypothalamic relay: a central control of bone mass. Cell..

[29] Hamrick MW, Pennington C, Newton D, Xie D, Isales C (2004). Leptin deficiency produces contrasting phenotypes in bones of the limb and spine. Bone..

[30] Wei Y, Wang L, Clark JC, Dass CR, Choong PF (2008). Elevated leptin expression in a rat model of fracture and traumatic brain injury. J Pharm Pharmacol.

[31] Yan H, Zhang HW, Fu P, Liu BL, Jin WZ, Duan SB, Xue J, Liu K, Sun ZM, Zeng XW (2013). Leptin's effect on accelerated fracture healing after traumatic brain injury. Neurol Res.

[32] Seemann R, Graef F, Garbe A, Keller J, Huang F, Duda G, Schmidt-Bleek K, Schaser KD, Tsitsilonis S (2018). Leptin-deficiency eradicates the positive effect of traumatic brain injury on bone healing: histological analyses in a combined trauma mouse model. J Musculoskelet Neuronal Interact.

[33] Garbe A, Graef F, Appelt J, Schmidt-Bleek K, Jahn D, Lunnemann T, et al. Leptin mediated pathways stabilize posttraumatic insulin and Osteocalcin patterns after long bone fracture and concomitant traumatic brain injury and thus influence fracture healing in a combined murine trauma model. Int J Mol Sci. 2020;21(23). 10.3390/ijms21239144. 10.3390/ijms21239144 PMC7729898 33266324

[34] Wildburger R, Zarkovic N, Tonkovic G, Skoric T, Frech S, Hartleb M, Loncaric I, Zarkovic K (1998). Post-traumatic hormonal disturbances: prolactin as a link between head injury and enhanced osteogenesis. J Endocrinol Investig.

[35] Zhang D, Zhang P, Wang Y, Han N, Tang C, Jiang B (2009). The influence of brain injury or peripheral nerve injury on calcitonin gene-related peptide concentration variation and fractures healing process. Artif Cells Blood Substit Immobil Biotechnol.

[36] Zhang JY, Yan GT, Liao J, Deng ZH, Xue H, Wang LH, Zhang K (2011). Leptin attenuates cerebral ischemia/reperfusion injury partially by CGRP expression. Eur J Pharmacol.

[37] Song Y, Bi L, Zhang Z, Huang Z, Hou W, Lu X, Sun P, Han Y (2012). Increased levels of calcitonin gene-related peptide in serum accelerate fracture healing following traumatic brain injury. Mol Med Rep.

[38] Clark D, Nakamura M, Miclau T, Marcucio R (2017). Effects of aging on fracture healing. Curr Osteoporos Rep.

[39] Shiu HT, Leung PC, Ko CH (2018). The roles of cellular and molecular components of a hematoma at early stage of bone healing. J Tissue Eng Regen Med.

[40] Whelan DB, Bhandari M, Stephen D, Kreder H, McKee MD, Zdero R, Schemitsch EH (2010). Development of the radiographic union score for tibial fractures for the assessment of tibial fracture healing after intramedullary fixation. J Trauma.

